# Salidroside Attenuates Hydrogen Peroxide-Induced Cell Damage Through a cAMP-Dependent Pathway

**DOI:** 10.3390/molecules16043371

**Published:** 2011-04-19

**Authors:** Shuang Guan, Wei Wang, Jing Lu, Wenhui Qian, Guoren Huang, Xuming Deng, Xuelin Wang

**Affiliations:** 1 Institute of Zoonoses, College of Animal Science and Veterinary Medicine, Jilin University, Changchun, Jilin 130062, China; E-Mails: gshuang1973@126.com (S.G.); wangwei@jluhp.edu.cn (W.W.); 2 Laboratory of Nutrition and Function Food, Jilin University, Changchun, Jilin 130062, China; E-Mail: xlujing1@yahoo.com.cn (J.L.)

**Keywords:** salidroside, antioxidant, AMP, cGMP, [Ca^2+^]i, ROS

## Abstract

Salidroside, a major component of *Rhodiola rosea L.*, has shown various pharmacological functions, including antioxidant effects, but the signal transduction pathway of its antioxidant effects is not very clear. In this study, we found that salidroside could attenuate hydrogen peroxide (H_2_O_2_)-induced HL-7702 cell damage, inhibit H_2_O_2_-induced cytosolic free Ca^2+^ ([Ca^2+^]i) elevation, scavenge reactive oxygen species (ROS) and increase 3’-5’-cyclic adenosine monophosphate (cAMP) level in a dose-dependent manner, but it couldn’t influence 3’-5’-cyclic guanosine monophosphate (cGMP) levels. Therefore, these results indicated that the antioxidant effects of salidroside were associated with down-regulation of [Ca^2+^]i, ROS occur via a cAMP-dependent pathway.

## 1. Introduction

**Figure 1:**
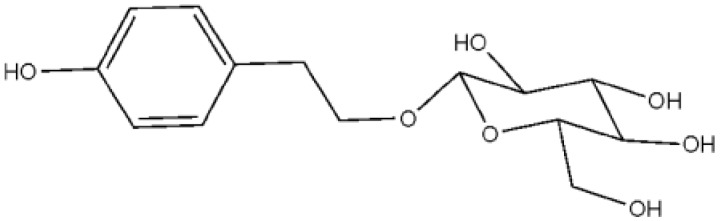
Structure of salidroside.

*Rhodiola rosea L.* has been widely used for a long time in Traditional Chinese Medicine. Salidroside (*р*-hydroxyphenethyl-β-D-glucoside, C_14_H_20_O_7_, 300.30), as shown in Figure 1, has been identified as the most potent ingredient in this medicinal plant [1]. Some studies have shown that salidroside possesses various pharmacological functions, including anti-aging [2], anti-cancer [3,4,5], anti-viral [6,7,8], anti-inflammatory [9], neuroprotective [10,11], hepatoprotective [9], cardiomyocytes protective [12,13], anti-diabetic [14] and antioxidant effects [15,16].

Oxidative damage, mediated by reactive oxygen species (ROS) has been thought as a major cause of atherosclerosis, cancer, liver disorder and the aging process [17]. In previously studies, people found that the antioxidant mechanism included [Ca^2+^]i and NO signaling pathways [18,19], thus it might involve the 3’-5’-cyclic adenosine monophosphate (cAMP)/3’-5’-cyclic guanosine monophosphate (cGMP)-dependent signaling pathway. To the best of our knowledge, no one has investigated the role of cAMP/cGMP in the antioxidant effects of salidroside, so the objective of the present paper was to examine the effects of salidroside on H_2_O_2_-induced oxidative damage, cAMP, cGMP; [Ca^2+^]i and ROS levels in HL-7702 cells to determine whether the cAMP/cGMP pathway participate in the antioxidant effects of salidroside. Our results would be benefitial for development of salidroside as a potential agent for treatment of oxidative stress-related diseases.

## 2. Results and Discussion

### 2.1. Protective Effect of Salidroside on H_2_O_2_-Induced Cell Damage

**Figure 2:**
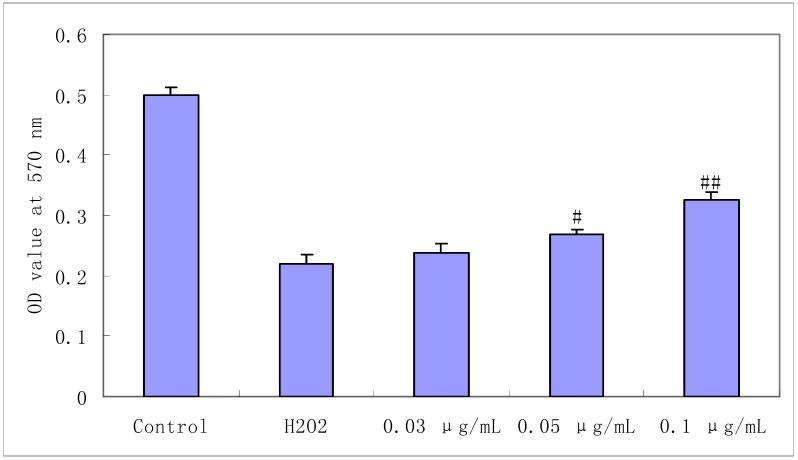
Protective effect of salidroside on H_2_O_2_-induced cell damage. Cells were treated with salidroside followed by H_2_O_2_ as described in 2.2 and cell viability was determined by MTT assay.

The protective effect of salidroside on H_2_O_2_-induced cell damage as determined by the MTT assay is shown in Figure 2. Incubating with H_2_O_2_(0.5 mM) for 100 min induced significant cell damage. Pretreatment with salidroside (0.03, 0.05, 0.1 μg/mL) could attenuate H_2_O_2_-induced cell damage in a dose-dependent manner (p < 0.05), though it couldn’t restore the damaged cells totally. Data are expressed as mean ± S.D. (n = 4). ^#^ p < 0.05 and ^##^ p < 0.01 compared with Control.

### 2.2. Salidroside Inhibited H_2_O_2_-Induced [Ca^2+^]i Elevation

**Figure 3:**
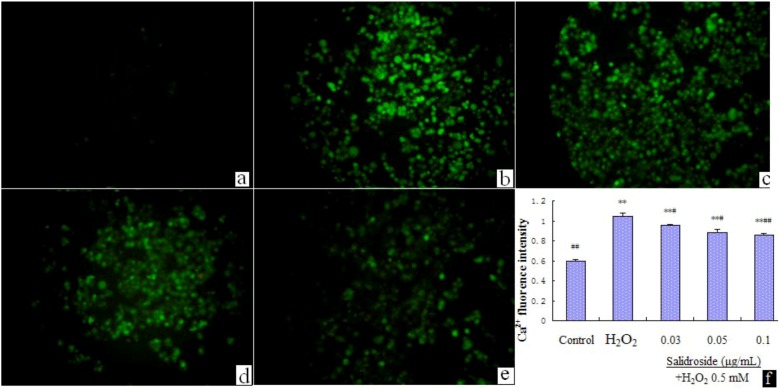
Salidroside inhibited H_2_O_2_-induced [Ca^2+^]i elevation (Recorded by inverted fluorescence microscope, Motic AE31 200×). (**a**). Control; (**b**). H_2_O_2_; (**c**). 0.03 μg/mL salidroside + H_2_O_2_; (**d**). 0.05 μg/mL salidroside + H_2_O_2_; (**e**). 0.1 μg/mL salidroside + H_2_O_2_; (**f**). Statistical analysis of fluorescence intensity data with Motic Advanced Images 3.2 software (China). Cells were treated with salidroside followed by H_2_O_2_ as described in 2.2. and [Ca^2+^]i levels were determined with Fluo-3/AM fluorescent dye.

We examined the effect of salidroside on [Ca^2+^]i. We found that incubating with H_2_O_2_(0.5 mM) for 100 min induced a significant [Ca^2+^]i elevation, while pre-incubation with salidroside dose dependently suppressed the elevation of [Ca^2+^]i (Figure 3). Data are expressed as mean ± S.D. (n = 4). * p < 0.05 and ** p < 0.01 compared with Control; # p < 0.05 and ## p < 0.01 compared with H_2_O_2_ group. 

### 2.3. Effects of Salidroside on the Formation of cAMP and cGMP

**Figure 4:**
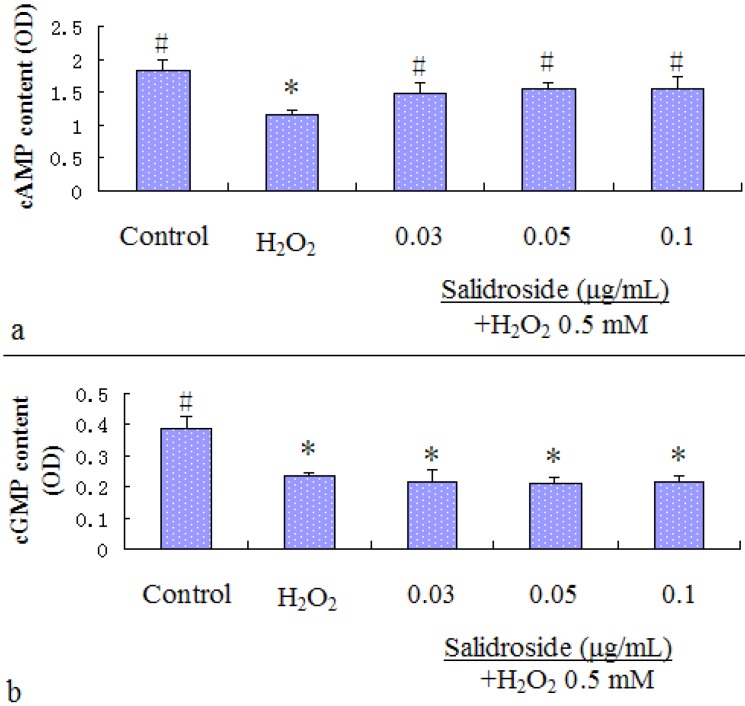
(**a**). Effect of salidroside on cAMP level in cells; (**b**). Effect of salidroside on cGMP level in cells. Cells were treated with salidroside followed by H_2_O_2_ as described in 2.2.Contents of cAMP and cGMP were measured using human cyclic adenosine monophosphate Elisa kits and human cyclic guanosine monophosphate Elisa kits respectively.

We investigated the effects of salidroside on the level of cAMP/cGMP. As shown in Figure 4, H_2_O_2_ decreased the levels of cAMP (Figure 4a) and cGMP (Figure 4b) in HL-7702 cells. The decreased level of cAMP was restored by treatment with salidroside, while the decreased level of cGMP was not changed. It showed that salidroside exerts antioxidant effects by cAMP, not cGMP. Data are expressed as absorbance density (OD) and presented as mean ± S.D. (n = 4). * p < 0.05 and ** p < 0.01 compared with Control; # p < 0.05 and ## p < 0.01 compared with H_2_O_2_ group.

### 2.4. Salidroside Inhibited H_2_O_2_-Induced ROS Elevation

**Figure 5:**
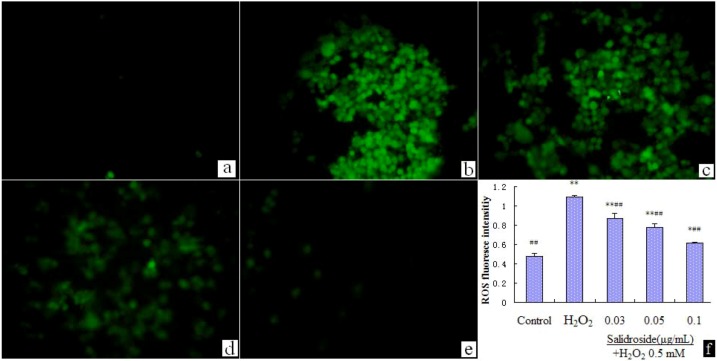
Salidroside inhibited H_2_O_2_-induced ROS elevation (Recorded by inverted fluorescence microscope, Motic AE31 200×). (**a**). Control; (**b**). H_2_O_2_; (**c**). 0.03 μg/mL salidroside + H_2_O_2_; (**d**). 0.05 μg/mL salidroside + H_2_O_2_; (**e**). 0.1 μg/mL salidroside + H_2_O_2_; (**f**). Statistical analysis of fluorescence intensity data with Motic Advanced Images 3.2 software. Cells were treated with salidroside followed by H_2_O_2_ as described in 2.2.

We examined the effect of salidroside on intracellular ROS. We found that incubating with H_2_O_2_(0.5 mM) for 100 min induced ROS elevation significantly, while pre-incubation with salidroside dose dependently suppressed the elevation of ROS (Figure 5). Statistical analysis of fluorescence intensity data with Motic Advanced Images 3.2 software. Data were expressed as mean ± S.D. (n = 4). * p < 0.05 and ** p < 0.01 compared with Control; # p < 0.05 and ## p < 0.01 compared with H_2_O_2_ group. 

### 2.5. Discussion

Today, oxidative damages caused by ROS and oxidative stress play important roles in many diseases, such as endocrine disruption [18]. H_2_O_2_, a major source of ROS, has been used in many studies to trigger oxidative damage [19]. In order to further studying antioxidant mechanism of salidroside, we investigated the effects of salidroside on H_2_O_2_-induced oxidative damage, cAMP, cGMP; [Ca^2+^]i and ROS level in HL-7702 cells.

In this paper, we used H_2_O_2_ 0.5 mM to trigger oxidative damage in HL-7702 cells. Incubating with H_2_O_2_ for 100 min induced significant cell damage. Salidroside (0.03, 0.05, 0.1 μg/mL) could attenuate H_2_O_2_-induced damage in a dose-dependent manner. It is suggested that salidroside at dose of 0.03, 0.05, 0.1 μg/mL could efficiently protect cells from oxidative damage. 

There is a mutual relationship between calcium and ROS signaling, which affects the local Ca^2+^ homeostasis [20]. H_2_O_2_ induced an [Ca^2+^]i rise which could still be evoked upon [Ca^2+^]i withdrawal and mitochondrial uncoupling [21]. In our research, 0.5 mM H_2_O_2_ successfully increased the level of [Ca^2+^]i in HL-7702 cells. [Ca^2+^]i levels are critical to cell life and changes in [Ca^2+^]i will affect matrix synthesis, as well as other functions [22]. In this experiment, salidroside inhibited H_2_O_2_-induced [Ca^2+^]i and ROS level and might be used in Ca^2+^ overloading disease.

cAMP is one of the most important second messengers in regulating a wide variety of cellular events and processes, such as metabolism, gene expression, cell division, migration, exocytosis and secretion. In non-excitable cells the fluctuations of [Ca^2+^]i and [cAMP]i are often interrelated and linked via adenylate cyclase or phosphodiesterase [23]. Elevation of the cAMP level will decrease the [Ca^2+^]i, and our result is consistent with this. The increased level of cAMP participates in activating PKA and consequently this enzyme phosphorylates its substrate proteins. Thus, the increased level of cAMP is linked to the activation of PKA [24]. Salidroside influenced H_2_O_2_-induced cAMP level, so it might be linked PKA-mediated phosphorylation.

cGMP is an important secondary messenger synthesized by the guanylyl cyclases and is also included in many cellular events and processes. It is believed that cGMP is produced via the activation of guanylate cyclase in the presence or absence of NO. NO is produced by the activation of NOS and affect the formation of cGMP. cGMP could affect [Ca^2+^]_i_ through direct inhibition of L-type Ca^2+^ channels [15]. cAMP and cGMP both are regulators of NO [25]. Salidroside couldn’t influence cGMP level, so the change of NO may be mediated via cAMP-depend pathway.

In the experiment, we found that salidroside attenuated H_2_O_2_-induced cell damage, inhibited H_2_O_2_-induced [Ca^2+^]i elevation, scavenged ROS at low dose through cAMP-dependent pathway. These findings suggested that antioxidant protection of salidroside was associated with the cAMP-dependent pathway and decrease of [Ca^2+^]i and ROS levels.

## 3. Experimental

### 3.1. Materials

Salidroside (standard material, purity >98%) was purchased from the National Institute for the Control of Pharmaceutical and Biological Products. 3-[4,5-Dimethyl-2-thiazolyl]-2,5-diphenyl-2-tetrazolium bromide (MTT) was purchased from Sigma (USA). Fluo-3/AM was obtained from Dojin Laboratories (Japan). 5-(and-6)-Chloromethyl-2’,7’-dichlorodihydrofluorescein diacetate, acetyl ester (CM-H_2_DCFDA) was obtained from Invitrogen (Molecular Probe), USA. RPMI 1640 was obtained from Gibco (USA). Human cyclic guanosine monophosphate ELISA kit and Human cyclic adenosine monophosphate ELISA kit were obtained from Blue Gene (Shanghai, China). FBS was obtained from Hangzhou (China). All other reagents were from commercial suppliers and of standard biochemical quality.

### 3.2. Protective Effect of Salidroside on H_2_O_2_-Induced Cell Damage

The human hepatocyte HL-7702 was obtained from SGST (China). The protective effect of salidroside was assessed by the MTT assay. Briefly, cells seeded on 96-well culture plates at 5 × 10^4^/ well were incubated with salidroside for 24 h. Following exposure to H_2_O_2_ (0.5 mM) for 100 min, the MTT solution (10 μL, 5 mg/mL) was added into each well and made the final concentration 0.5 mg/mL, and then the plates were incubated for an additional 2 h. After the medium was removed, DMSO (100 μL) was added into each well before reading the microplates at 570 nm. Cell viability was expressed as absorbance density (OD).

### 3.3. Determination of [Ca^2+^]i

The [Ca^2+^]i was determined using Fluo-3/acetoxymethyl ester (Fluo-3/AM), described previously with slight modification [26]. Briefly, cells were loaded for 20 min at 37 °C with 20 μM Fluo-3/AM containing 1 μM pluronic acid F-127 for proper dispersal and 0.25 mM sulfinpyrazone, to inhibit the leakage of the Fluo-3 dye. Shortly before use, a sample of loaded cells was washed with Krebs-Ringer-Hepes (KRH) buffer (130 mM NaCl, 1.3 mM KCl, 2.2 mM CaCl_2_, 1.2 mM MgSO_4_, 1.2 mM KH_2_PO_4_, 10 mM Hepes, 10 mM glucose, pH 7.4), to remove nonhydrolyzed Fluo-3/AM. Fluorescence measurements were performed using an inverted fluorescence microscope (Motic AE31invert, Xiamen, China).

### 3.4. Measurement of cAMP and cGMP

Cells (5 × 10^4^ /mL) were pre-incubated with various concentrations of salidroside for 48 h at 37 °C in 24-well culture plates, then incubated with H_2_O_2_ for 100 min. cAMP and cGMP were measured using the ELISA kits according to the manufacturer’s instructions. The levels of cAMP and cGMP were expressed by absorbance density (OD). 

### 3.5. Measurement of ROS

The intracellular ROS levels of cells were determined using CM-H2DCFDA assay [26]. For these experiments, cells were incubated with phosphate-buffered saline (PBS) and 5 mM CM-H2DCFDA. As a control, cells were incubated with PBS only. As a positive control, cells were incubated with PBS, 5 mM CM-H2DCFDA, and 0.5 mM H_2_O_2_. After a 100 min incubation period with 0.5 mM H_2_O_2_, cells were trypsinized, washed, and resuspended in PBS. The levels of fluorescence intensity were immediately detected using an inverted fluorescent microscope (Motic AE31invert, Xiamen, China).

### 3.6. Statistical Analysis

All data were shown as means ± S.D. (standard deviation of the mean) Statistical analyses were performed using Student’s *t*-test and one-way analysis of variance (ANOVA). p < 0.05 was considered as statistically significant.

## 4. Conclusions

The data reported here firstly suggested that the antioxidant effects of salidroside were associated with down-regulation of [Ca^2+^]i, ROS and at least partly via cAMP-dependent pathway.
